# The antidepressant effect of combined extracts of *Hypericum perforatum* and *Echium amoenum* supplementation in patients with depression symptoms: A randomized clinical trial 

**DOI:** 10.22038/AJP.2023.21707

**Published:** 2023

**Authors:** Akram Sadeghi, Fatemeh Ghorayshi, Hojjat Baghshahi, Hossein Akbari, Mohammad Reza Memarzadeh, Mohsen Taghizadeh, Alireza Safaei

**Affiliations:** 1 *Department of Psychiatry, Kashan University of Medical Sciences, Kashan, Iran*; 2 *Barij Medicinal Plants Research Center, Kashan, Iran *; 3 *Social Determinants of Health Research Center, Department of Biostatistics and Epidemiology, Kashan University of Medical Sciences, Kashan, Iran*; 4 *Research Center for Biochemistry and Nutrition in Metabolic Disease, Kashan University of Medical Sciences, Kashan, Iran*

**Keywords:** Anti-Anxiety Drug, Fluoxetine, Depression, Herbal drug, Traditional Medicine

## Abstract

**Objective:**

*Echium amoenum* and *Hypericum perforatum* dried flowers have been used for therapy of mental disorders in Iranian traditional medicine. In this study, we assessed the efficacy of the *E. amoenum* and *H. perforatum* extracts in patients with mild to moderate depression.

**Materials and Methods:**

In an 8-week double-blind, parallel-group trial, 51 patients randomly consumed 20 mg of fluoxetine or 350 mg of herbal medicine twice daily. The Hamilton Rating Scale for Depression (HAM-D) was used to assess depression severity in patients at weeks 0, 4, and 8.

**Results:**

According to the Hamilton score, there were no significant differences between the fluoxetine- and herbal medicine-treated groups after 4 and 8 weeks (p>0.05). Dry mouth was the only reported side effect which was significantly lower in the herbal group (p<0.05) in weeks 2 and 4.

**Conclusion:**

*E. amoenum* and *H. perforatum* have anti-depressant properties similar to fluoxetine and they can be used to treat depression as an alternative to fluoxetine.

## Introduction

Depression is one of the most common mental illnesses, posing a heavy burden on the society (Godfrin and Van Heeringen, 2010; Sadeghi et al., 2010; Sooky et al., 2010). 

According to the World Health Organization (WHO), depression was the fourth leading cause of disease burden (WHO, 2001). It is projected to become the second leading cause of death by 2030 (Mohammadi et al., 2014; Yaghubi et al., 2017). In other words, major depressive illness has a significant economic, emotional, and social impact on individuals, their families, and the community (Klainin-Yobas et al., 2012; Sooky et al., 2010). Data shows that the incidence of this disease and the consequent decline in quality of life are growing. Studies reveal that around one million lives are lost yearly due to suicide, equating to 3,000 suicide deaths every day. For each suicide that leads to death, there are about twenty suicide attempts (Marcus et al., 2012; Shahidi et al., 2017).

In addition to having adverse effects, drug therapies for depression are inadequate (Bech et al., 2000). A meta-analysis of 35 studies found that the difference between antidepressant and placebo responses in patients with depression was not clinically significant, and the difference was only significant in cases of severe depression. This pattern is attributable to a decrease in placebo effects rather than an increase in pharmaceutical efficacy (Kirsch, 2015). 

Most of these medications have anticholinergic effects, and cause hypotension, and arrhythmias as adverse effects. As a result, affordable, more effective, and less harmful drugs are required. However, in the treatment of depression, the main goal is to improve efficacy while minimizing unpleasant events during acute times and keeping the patient in the desired condition and stability (Akhondzadeh et al., 2003). Herbal medications have recently been considered in the therapy of many mental illnesses (Desai and Grossberg, 2003; LaFrance Jr et al., 2000) since they have fewer adverse effects and may be administered alone or in combination (Akhondzadeh, 2007).

*Hypericum perforatum*, also known as St. John's wort, is a medicinal plant used to treat depression (Stevinson and Ernst, 1999). This extract was comparable to synthetic antidepressants such as amitriptyline (Schulz, 2002). Hyperforin and hypericin are the most important compounds of this plant (Saddiqe et al., 2010). The quality of medicinal products based on *H. perforatum* is standardized with hypericin content (Hevia et al., 2002). Mechanisms expressed for the antidepressant effects of this plant include (1) non-selective inhibition of reabsorption of serotonin, noradrenaline, and dopamine, which increases serotonergic and dopaminergic receptors, (2) increased affinity for gamma-aminobutyric acid (GABA) receptors and (3) inhibition of monoamine oxidase activity (Rodriguez-Landa and Contreras, 2003).

*Echium amoenum* is also one of the most widely utilized herbs for treating ailments and mental problems. The aqueous extract of this plant is an effective and safe therapy for severe depressive illness (Sayyah et al., 2006). In mice, *E. vulgare* flower had an effect similar to imipramine, which elevated neurotransmitters such as norepinephrine and serotonin levels (Moallem et al., 2007). The seeds of this plant are also rich in omega-3 and omega-6 essential fatty acids (Baker et al., 2016) which have the potential to prevent many neurological diseases such as anxiety, depression, Alzheimer disease, and seizure (Nouri et al., 2019).

For the first time, we studied the effects of combined extracts of *H. perforatum* and *E. amoenum* in patients with depressive symptoms compared with standard treatment (fluoxetine).

## Materials and Methods

**Study participants, and inclusion and exclusion criteria**

This study was conducted in Kargarnejad Hospital in Kashan, Iran, from June 2017 to March 2018. Fifty-one individuals between 18 to 55 years old with mild to moderate depression and no other psychiatric problems or antidepressants use participated. Patients were included in the trial if they met the following criteria: age range of 18–55 years; having mild to moderate depression based on the Hamilton Anxiety Rating Scale and Structured Clinical Interview for DSM Disorders for DSM-IV-TR, and interested in participating in the study. Participants were excluded if they had at least one of the following conditions during the intervention: pregnancy or lactation; allergy to the studied medicinal plants; existence of a cognitive disorder in the past year; history of bipolar disorder (Based on SCID-I Interview); use of psychotropic drugs in the past two weeks; suffering from severe medical diseases; use of psychedelics; patients with suicidal ideation; taking or use history of vitamin supplements in the past two months; psychotic disorders or refusal to continue taking the herbal supplement. 

**Ethical considerations**

The trial was reviewed, approved, and monitored by the Ethics Committee of Kashan University of Medical Sciences and Health Services (Ethical Code: IR.KAUMS.REC.1396.46). Furthermore, the trial was registered by the Iranian Registry of Clinical Trials with the following code: IRCT2017082612438N24. All of the participants signed an informed consent form before enrollment in the study.

**Sample size**

According to a previous study, the mean and standard deviation of depression score reduction based on the HAM-D criterion in Hypericum group was equal to 8.68±0.68, while this value was 10.53±0.72 in the sertraline group (Hypericum Depression Trial Study Group, 2002). The minimum needed sample in each group was determined to be 16 people based on the 95% power, 95% confidence level, and the maximum negligible error of one unit assuming non-inferiority.

**Study design**

We conducted a double-blinded randomized trial on patients referred to psychiatric clinics affiliated with Kashan University of Medical Sciences, Kashan, Iran. Study process was described to each participant and written informed consent was obtained from all patients before entering the study. They also learned about their rights to deny participation and quit the study. 

The Hamilton (Hamilton, 1986) and personal information questionnaires were completed by the psychologist and the patients, respectively. A personality questionnaire contains gender, age, weight, height, education, occupation, marital status, physical or mental disease history, and use history of psychiatric drugs. The Hamilton questionnaire assesses the severity of depression by a therapist, consists of 24 questions and covers different dimensions of depression (behavioral, physical, cognitive, emotional, guilt, hypochondria, sexual issues, work, suicide, and sleep disorders). According to the questionnaire, a score of 17-24 shows mild and 25-30 for moderate depression (Hamilton, 1960). The eligible patients were randomly assigned into two groups. One group received two capsules of 350 mg of the herbal drug daily for eight weeks, and the other group received two capsules of fluoxetine 20 mg daily for eight weeks. Following the start of the trial, the patients were visited twice in four weeks to review the Hamilton criteria, exclusion criteria, and possible problems (nausea, vomiting, headache, dizziness, stomach discomfort, and dry mouth). If there was no difference in the patient's Hamilton criteria after two weeks, the patient was excluded from the study after a clinical interview by a psychiatrist based on DSM-IV criteria.

**Plant extracts**

The voucher specimens of *E. amoenum* and *H. perforatum* have been deposited in the Herbarium of the Agriculture Department of Barij Essence Pharmaceutical Company under numbers 200-1 and 2003-1, respectively. One hundred grams of *E. amoenum* was added to 3 L of boiling distilled water. The mixture was stirred and boiled for 10 min. Then, the solution was cooled to room temperature. After filtration, 1 L of 96% ethanol was mixed with 2 L of the extract to prevent microorganism growth during drying at 50°C.

*H. perforatum* liquid extract was obtained with 1000 g of crushed dried *H. perforatum* in 3000 ml of 70% ethanol. The extraction lasted 48 hr. After that, the stock solution was filtered through a filter paper. The 70° ethanol solvent was removed as much as possible from the extract under reduced pressure and the concentrated extract was obtained.

Then the concentrated extract was dried with a spray dryer and stored in a refrigerator and under a dehumidifier to produce capsules.

**Preparation of drugs**

The herbal capsule supplement contained 100 mg of the dried extract of the flowers of *E. amoenum* based on 60 mg of total phenolic contents, and 250 mg of the dried extract of the aerial parts of *H. perforatum* (total hypericin 0.25 mg/ml). After processing the herbal compound, random codes were provided to the project executors in sealed envelopes using a random-number table with a permuted block design (block size of 4).

**Randomization**

Seventy-four eligible patients were allocated randomly to either the herbal medicine or fluoxetine groups through computer-generated random numbers. All participants and investigators were blind to the allocation of the patients. Herbal medicine and fluoxetine capsules were similar in size, weight, shape, and color. Additionally, pill bottles were identical. The type of medicine allocated to each group was unknown to patients, physicians, drug delivery personnel, and data analysts, except pharmacists.

**Statistical methods**

After collecting information, the effect of each group was evaluated using a Wilcoxon and paired t-test. Based on the normality of the data, differences between groups were analyzed using an independent t-test and Mann-Whitney U. Finally, the repeated measure analysis of the variance was done using multivariate effects of treatment groups.

## Results

**Patients enrollment**

**Figure 1:**
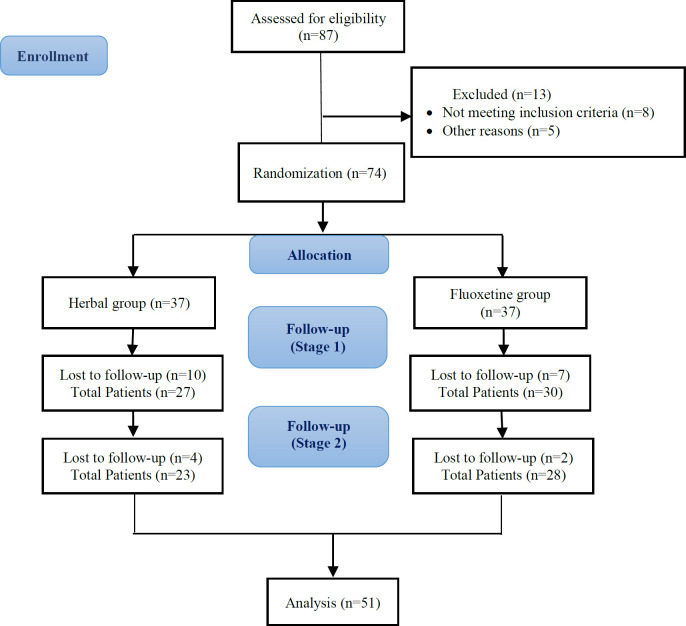
Consort flow diagram of the study

Out of the 87 eligible patients, thirteen were excluded from the study because they did not meet the inclusion criteria or for other reasons. The remaining 74 patients were randomly assigned to herbal medicine group (n=37) or fluoxetine group (n=37). The final analysis included data from 23 herbal medicine participants and 28 fluoxetine participants (Figure 1). 

**Baseline clinical characteristics**

Finally, this research involved 51 individuals aged 11 to 58 (28 females and 38 males; mean age of 35.10±8.05 years). At the commencement of the study, there was no significant difference between the two groups in terms of demographics, history of mental disorder, drug use, or Hamilton score (Table 1).

**Clinical response**

At the start, first, and second months following the study, Hamilton's average scores were 27.64, 20.64, and 16.93 in the fluoxetine group, and 27.22, 21.13, and 17.0 in the herbal medication group, respectively. The difference between the two groups was not significant during the study (p>0.05, Tables 2 and 3). The comparison between the groups showed that there was no statistical difference between the two groups at the beginning of the study, the second and the fourth week. 

**Table1:** Demographic and clinical characteristics of the patients in herbal and fluoxetine groups at the beginning of the study

**Variables**	**Status**	**Groups**		**p-value**
		**Fluoxetine** **n (%)**	**Herbal group** **n (%)**	
**Gender**	Female	16 (57.1)	12 (52.2)	0.723*
	Male	12 (42.9)	11 (47.8)	
**Age (year)**	$\overset{\text{®}}{X}$ *±* SD	36.8±11.15	34.7±8.65	0.473**
**Marital status**	Married	16 (57.1)	12 (52.2)	0.723*
	Single	12 (42.9)	11 (47.8)	
**Education**	primary education	11 (39.2)	5.0 (21.7)	0.329*
	Middle and high school	13 (46.4)	12 (52.4)	
	University	4.0 (14.3)	6.0 (26.1)	
**Job**	Unemployed	5.0 (17.9)	4.0 (17.4)	0.445*
	Employed	10 (35.7)	12 (52.2)	
	Housekeeper	13 (46.4)	7.0 (30.4)	
**Height (cm)**	$\overset{\text{®}}{X}$ *±* SD	172.04 05.95	173.61±5.93	0.351**
**Weight (kg)**	$\overset{\text{®}}{X}$ *±* SD	72.07±11.39	75.17±8.15	0.279**
**Getting sick **		13 (64.4)	9.0 (39.1)	0.601*
**Taking medication**		10 (35.7)	8.0 (34.8)	0.945*
**History of mental disorder**		11 (39.3)	8.0 (34.8)	0.741*
**Taking psychotropic drugs**		5.0 (17.9)	6.0 (26.1)	0.477*
**Hamilton score**		27.64±2.57	27.22±2.94	0.584*

*Chi- Square test. ** Independent T-test

**Figure 2:**
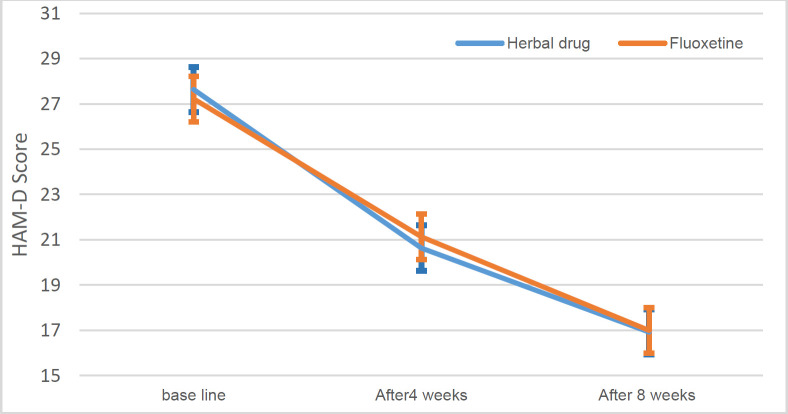
The mean and 95% confidence interval of Hamilton score in the herbal and fluoxetine groups during the study

The effect of the time in repeated measures ANOVA was significant (p<0.001), which showed a significant decrease in Hamilton score in both groups. The time-group interaction effect was not significant, indicating the same score changes between the groups over time (p=0.486, Table 2 and Figure 2).

In the first two weeks of medication, 50% of fluoxetine patients and 30.4% of herbal patients experienced side effects. The most prevalent side effect throughout this time was dry mouth, which was significantly more common in the fluoxetine group (p=0.007). However, there was no significant difference in the occurrence of other complications between the two study groups. After the fourth week, dry mouth was still the most common complication in the fluoxetine group (p=0.013). From week six through the completion of the study, there was no significant difference in side effects between the two study groups (Table 4).

**Table 2:** Mean and standard deviation of Hamilton score in the herbal and fluoxetine groups during the study

**Period of study**	**Fluoxetine**		**Herbal group**	**p** ^3^
Start of the study	27.64±2.57		27.22±2.94	0.584
4 weeks after the study	20.64±3.14		21.13±3.75	0.615
8 weeks after the study	16.93±3.36		17.00±3.25	0.939
The effect of time (P^1^)		<0.001		
The effect of time × group (P^2^)		0.486		

^1^ Effect of time in repeated measure analysis of variance. ^2^ Effect of time×group in repeated measure analysis of variance. ^3^ Independent T-test

**Table 3:** Frequency of treatment continuation in the herbal and fluoxetine groups during the study

**Time**	**Fluoxetine**	**Herbal group**	**p- Value**
After 2 weeks	27 (96.9)	22 (95.7)	NS**
After 4 weeks	27 (96.4)	21 (91.3)	0.162*
After 6 weeks	26 (92.9)	21 (91.3)	NS**
After 8 weeks	25 (89.3)	23 (100)	0.242*

* Chi- Square test. ** Fisher's exact test

**Table 4:** Frequency of side effects in the herbal and fluoxetine groups during the study

**Time**	**Complication**	**Fluoxetine**	**Herbal group**	**p- Value**
After 2 weeks	Existence of complications	14 (50)	7.0 (30.4)	0.158*
	Nausea	2.0 (7.1)	4.0 (17.4)	0.390**
	Dry mouth	12.0 (42.9)	4.0 (8.7)	0.007*
	Heart problems	8.0 (28.6)	7.0 (30.4)	0.884*
	Other complications	4.0 (14.3)	3.0 (10.7)	NS**
After 4 weeks	Existence of complications	10 (35.7)	5.0 (21.7)	0.276*
	Nausea	3.0 (10.7)	2.0 (8.7)	NS**
	Dry mouth	11 (39.3)	2.0 (8.7)	0.013**
	Physical problems	7.0 (25)	5.0 (21.7)	0.785*
	Other complications	2.0 (7.1)	2.0 (4.3)	NS**
After 6 weeks	Existence of complications	4 (14.3)	3.0 (13)	NS**
	Nausea	1.0 (3.6)	1.0 (4.3)	NS**
	Dry mouth	4.0 (14.3)	1.0 (4.3)	0.362**
	Physical problems	7.0 (25)	6.0 (26.1)	NS*
	Other complications	1.0 (3.6)	2(8.7)	0.495**
After 8 weeks	Existence of complications	2 (7.1)	2.0 (8.7)	NS**
	Nausea	-	1.0 (4.3)	0.451**
	Dry mouth	4.0 (14.3)	1.0 (4.3)	0.362**
	Physical problems	6.0 (21.4)	5.0 (21.7)	0.979*
	Other complications	1.0 (3.6)	-	NS**

Other complication included headache, vertigo, stomachache, physical problems including diarrhea, constipation, indigestion, weight loss, and loss of appetite. * Chi- Square test. ** Fisher’s exact test. N.S: not significant.

## Discussion

There was no significant difference in Hamilton scores between the herbal and fluoxetine groups in the present study. However, Hamilton scores in both groups reduced as intervention time progressed, indicating that both herbal medicine and fluoxetine treatments had therapeutic effects. Among the complications, dry mouth was the only complication that was significantly more common in the fluoxetine group in the second and fourth weeks after starting treatment.

In 2005, Fava et al. conducted a 12-week trial comparing the effects of 900 mg/d *H. perforatum* extract and fluoxetine on major depression (Fava et al., 2005). Their results showed that this plant is more effective than fluoxetine in treating depression. The higher effectiveness of H. perforatum extract in their study may be due to long-term use, higher doses of the herbal compound, or lower doses of fluoxetine. Bjerkenstedt et al. also showed that *H. perforatum* is much better tolerated than fluoxetin (Bjerkenstedt et al., 2005). However, they reported that neither *H. perforatum* nor fluoxetine was more effective than placebo in the short-term treatment of mild to moderate depression.

Another study found that *H. perforatum* extract was as effective as paroxetine in treating moderate to severe depression and was better tolerated (Szegedi et al., 2005). The findings of 34 investigations comparing the effects of *H. perforatum* extract on depressive disorders with synthetic drugs revealed that this herb is as effective as synthetic pharmaceuticals in treating mild to moderate depression (Schulz, 2002). 

Gastpar et al. evaluated the effects of 612 mg of *H. perforatum* extract combined with 50 mg of sertraline for 12 weeks in patients with mild depression. After 12 weeks of therapy, both groups' Hamilton Depression Scale scores improved (Gastpar et al., 2005). This research found that hypericum and sertraline extracts have similar side effects.

Studies have confirmed the antidepressant properties of hypericin, the main active element in *H. perforatum*, and *E. amoenum* (Rodriguez-Landa and Contreras, 2003; Fava et al., 2005; Sayyah et al., 2006). Antidepressants in aqueous and alcoholic extracts of the aerial parts of *Echium vulgare* L. were also tested in mice (Moallem et al., 2007). Their research showed that the aqueous extract in low doses and ethanolic extract in high doses had an antidepressant effect. This plant may be effective on neurotransmitters like norepinephrine and serotonin, making it a potential therapeutic for treating depression. 

Phytochemical evaluation of *E. amoenum* extract showed that the plant contains saponins, flavonoids, unsaturated terpenoids, and sterols (Shafaghi et al., 2010). Quercetin as a flavonoid reduced the immobility period in diabetic rats compared to fluoxetine and imipramine (Anjaneyulu et al., 2003). 

The current study showed that the combination of *Hypericum perforatum* and *Echium amoenum* extracts is as effective as fluoxetine in treating mild to moderate depression. It was also well tolerated by the patients. Investigating these medicinal herbs' effects on the dopamine transporter, norepinephrine transporter, and serotonin transporter in animal research is recommended for a better understanding.

Because the follow-up period was long, we urged patients to maintain their therapy and attend the project at the start of the trial and intervals during the telephone and in-person follow-up.

## Conflicts of interest

The authors have declared that there is no conflict of interest.
